# Intestinal perforation in recurrent cervical cancer following bevacizumab and pembrolizumab therapy: A case report

**DOI:** 10.1097/MD.0000000000040473

**Published:** 2025-04-11

**Authors:** Yuanchun Fan, Shihao Liu, Jiangjing Zhao, Yawei Fu, Jiahui Yang, Chunyang Wang, Hui Zhang

**Affiliations:** a The Gynecology Department, The Fourth Hospital of Hebei Medical University, Shijiazhuang, Hebei, China.

**Keywords:** bevacizumab, cervical cancer, intestinal perforation, pembrolizumab

## Abstract

**Rationale:**

Since the advent of immunotherapy in clinical practice, it has profoundly transformed the paradigm of cancer treatment and has been rapidly adopted in clinical settings. Concurrently, the combination of immunotherapy with anti-angiogenic therapy has shown great promise in clinical research. The inevitable joint application brings about a greater number of adverse reactions. These adverse reactions are often perplexing, with the uncertainty of whether they stem from immunotherapy, anti-angiogenic therapy, or both. This is a case report of adverse reactions occurring when immune drugs and anti-vascular drugs are used together. This case is analyzed to provide a warning for adverse reactions in the clinical application of anti-angiogenic therapy combined with immunotherapy.

**Patient concerns:**

A 52-year-old cervical cancer patient with metastases had abdominal pain and fever post-treatment with bevacizumab, pembrolizumab, and chemotherapy, suggesting intestinal perforation.

**Diagnoses:**

After 2 chemotherapy cycles with bevacizumab and pembrolizumab, the patient had fever up to 39°C and abdominal pain. Exam showed tenderness, rigidity, and weak bowel sounds. Blood tests revealed leukocytosis and neutrophilia. Imaging indicated pneumoperitoneum and possible intestinal obstruction.

**Interventions:**

Emergency laparotomy revealed a small intestine perforation with strictures, leading to resection and ileostomy due to edema.

**Outcomes:**

The postoperative recovery was good. We consider intestinal perforation caused by bevacizumab. Therefore, the patient was subsequently discontinued from bevacizumab and continued to receive paclitaxel, cisplatin and pembrolizumab. At present, the patient has finished chemotherapy and is receiving pembrolizumab maintenance therapy with no significant gastrointestinal adverse reactions.

**Lessons:**

Anti-angiogenic drugs and immunotherapy drugs each have their own side effects, and the occurrence of adverse reactions becomes more complex when used in combination. In the clinical process of combined medication, more attention should be paid to adverse reactions, early identification of severe adverse reactions, and active management.

## 1. Introduction

Immunotherapy has emerged as a highly effective treatment for cancer and is now widely accepted in clinical practice. Immune checkpoint inhibitors (ICIs) are considered the most successful form of immunotherapy to date. In recent years, the FDA has approved ICIs for multiple clinical indications, primarily including programmed death-1/programmed death 1 ligand 1 (PD-1/PD-L1) inhibitors (such as pembrolizumab) and T-lymphocyte antigen 4 inhibitors. The use of ICIs has revolutionized the treatment of various malignant tumors. However, the current effectiveness is not ideal, with an objective response rate of approximately 15% to 25%,^[1–3]^ and even lower for some cancers. Therefore, in recent years, many studies have focused on developing combined treatment strategies that can activate anti-tumor immunity and enhance therapeutic effects. Evidence suggests that anti-angiogenic treatment enhances tumor immunity by normalizing tumor blood vessels, thereby improving the immune microenvironment. In the era of immunotherapy, the combination of anti-angiogenic drugs and immunotherapy has demonstrated a significant synergistic effect, effectively inhibiting tumor growth and metastasis.^[4]^ However, the benefits of enhanced anti-tumor efficacy must be weighed against the increased risk of toxicity.

We report a case of a patient with cervical cancer who relapsed after concurrent chemoradiotherapy and subsequently developed acute abdominal pain and fever during the combined immunotherapy with bevacizumab and pembrolizumab, with a clinical diagnosis of intestinal perforation. Surgical treatment was then performed. The patient discontinued bevacizumab therapy afterward. This case is analyzed to provide a warning for adverse reactions in the clinical application of anti-angiogenic therapy combined with immunotherapy.

## 2. Case summary

The patient is a 52-year-old female, 160 cm tall and weighing 46 kg, who presented to the Department of Gynecology at the Fourth Hospital of Hebei Medical University on November 1, 2020. Her primary complaint was “abdominal pain for 2 days,” following a history of “cervical squamous cell carcinoma stage IIB treated with radiotherapy and chemotherapy 4 years ago, with a relapse after 2 courses of chemotherapy.”

Four years prior, the patient had undergone radical radiotherapy and chemotherapy (external irradiation and intracavitary brachytherapy) for stage IIB cervical squamous cell carcinoma at the Gynecologic Oncology Department of the Fourth Hospital of Hebei Medical University, achieving complete remission (CR) at the end of treatment. She has been under regular follow-up since then.

**Figure 1.**
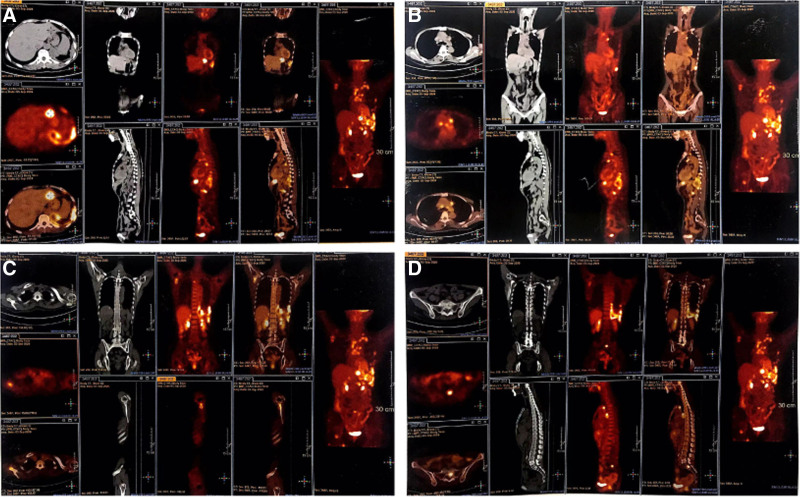
PET-CT high metabolic area. (A) Upper segment of left outer lobe of liver. (B) Multiple lymph node shadows on the left side of the abdominal aorta in mediastinum and retroperitoneum. (C) Left scapula. (D) Sacrum. PET-CT = positron emission tomography/computed tomography.

**Figure 2.**
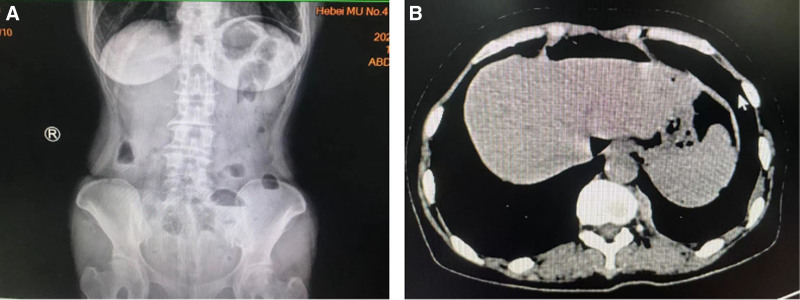
(A) Standing abdominal plain film. (B) Abdominal CT. CT = computed tomography.

On August 27, 2023, a reexamination revealed that the patient’s SCC had elevated to 61.1 ng/mL. A PET-CT scan (Fig. 1) revealed the following findings: A slightly low-density round lesion in the upper segment of the left outer leaf of the liver (S2) with abnormal glucose metabolism, suggested metastasis. Multiple lymph nodes in the mediastinum, retroperitoneal area, and left side of the abdominal aorta were enlarged, with abnormal glucose metabolism, suggested multiple lymph node metastases. Abnormal density in the medullary cavity of the left scapula, the right side of the vertebral arch of the third thoracic vertebra, the sacrum, and the inner side of the upper segment of the right femur, with abnormal glucose metabolism, suggested bone metastasis of cervical cancer. These findings indicated recurrent stage IIB cervical squamous cell carcinoma after radiotherapy and chemotherapy. The patient’s Combined Positive Score was > 1 score, and she began treatment on September 14, 2023, with a regimen of paclitaxel (175 mg/m²), cisplatin (50 mg/m²), bevacizumab (15 mg/kg), and pembrolizumab (200 mg) every 3 weeks. She received a total of 2 cycles of treatment, with the last dose administered on October 5, 2023. Following the treatment, she experienced increased bowel movements and loose stools, about 3 times a day, which improved with oral anti-diarrheal medication. On November 1, 2023, she suddenly developed abdominal pain accompanied by fever, with the highest recorded temperature at 39°C. Physical examination revealed obvious tenderness and muscle tension throughout the abdomen, without significant rebound tenderness. Bowel sounds were weak, and percussion was tympanitic. Blood tests showed: white blood cells at 10.47 × 10^9^/L, neutrophil percentage at 95%, and hemoglobin at 81 g/L. An erect abdominal plain film suggested pneumoperitoneum and multiple small liquid and gas shadows in the abdomen, raising the possibility of incomplete intestinal obstruction (Fig. 2). Preliminary diagnosis: abdominal pain of undetermined cause, possible gastrointestinal perforation; recurrent stage IIB cervical squamous cell carcinoma after radiotherapy and chemotherapy; status post 2 cycles of chemotherapy.

The patient exhibited clear symptoms and signs of peritonitis. Given her history of radiotherapy, chemotherapy, and bevacizumab use, the gastrointestinal surgery consultation determined a high likelihood of intestinal perforation. The risks and necessity of surgery were thoroughly explained to the patient and her family, who understood and consented to the procedure. An emergency laparotomy was performed on November 1, 2023.

**Figure 3.**
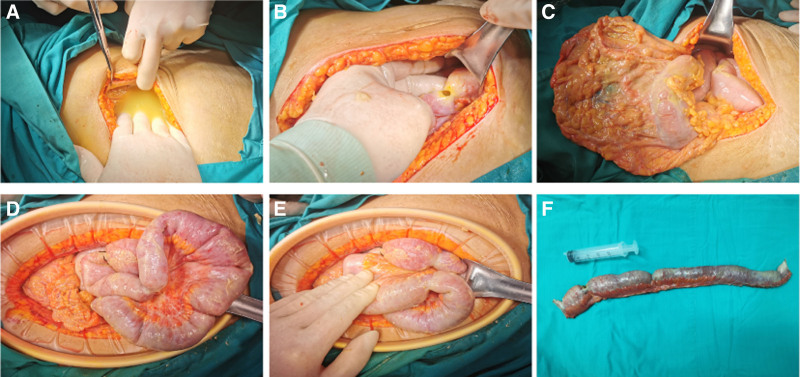
Intraoperative conditions. (A) Purulent abdominal effusion. (B) Perforation. (C) The omentum and bowel are full of pus and moss. (D) Intestinal dilatation edema. (E) Segmental stenosis of bowel. (F) Remove the bowel tube.

Intraoperative findings included a large amount of yellow purulent fluid in the abdominal cavity, approximately 800 mL. The greater omentum, liver, spleen, and intestinal surfaces were covered with purulent exudate. After separating the adhesions, a 0.5 cm perforation was identified in the small intestine, approximately 210 cm from the ligament of Treitz, with intestinal fluid leaking out. Multiple segmental narrowings were noted around the perforation. The intestine was significantly dilated and edematous. About 50 cm of the affected intestine, including the perforated and narrowed sections, was resected (Fig. 3). Due to the significant edema of the intestine, an ileostomy was performed.

Postoperative anti-inflammatory fluid and nutritional support treatment were given, and the postoperative pathology showed: chronic mucosal inflammation, edema with congestion under the mucosa, and ulcers and perforations were formed. The patient recovered well after the operation and was discharged smoothly 14 days after the operation.

We consider intestinal perforation caused by bevacizumab. Therefore, the patient was subsequently discontinued from bevacizumab and continued to receive paclitaxel, cisplatin and pembrolizumab. At present, the patient has finished chemotherapy and is receiving pembrolizumab maintenance therapy with no significant gastrointestinal adverse reactions.

## 3. Discussion

Bevacizumab is a recombinant humanized anti-vascular endothelial growth factor (VEGF) monoclonal antibody.^[5]^ VEGF is widely expressed in human tissues, and blocking the VEGF pathway can lead to various associated adverse reactions. Initially approved in 2004 as a first-line treatment for metastatic colorectal cancer, bevacizumab’s indications have expanded to include metastatic breast cancer, non-small cell lung cancer, glioblastoma, renal cell cancer, ovarian cancer, cervical cancer, and other solid tumors.^[6,7]^ Its role in cervical cancer has been well established.^[8,9]^ Gastrointestinal perforation is a rare but potentially fatal adverse reaction associated with bevacizumab, reported in various tumor treatments.^[10–13]^ The mortality rate of gastrointestinal perforation in patients treated with bevacizumab has been reported as high as 50%,^[14]^ with common sites of perforation including the colon, small intestine, and stomach. A systematic review indicated that the incidence of gastrointestinal perforation in patients receiving bevacizumab ranged from 0.3% to 3%.^[15]^ In the GOG240 trial, the incidence of gastrointestinal perforation related to bevacizumab was reported as 3%.^[8]^

Pembrolizumab is a PD-1 inhibitor, and common adverse reactions associated with PD-1 and PD-L1 blockers include pneumonitis, myalgia, hypothyroidism, gastrointestinal reactions, arthralgia, and vitiligo. Immune treatment-related gastrointestinal toxicity primarily manifests as lower gastrointestinal adverse events such as diarrhea and colitis/enteritis, occurring in 30% to 50% of cases. Upper gastrointestinal adverse events like nausea and vomiting occur in approximately 36% of cases. Studies indicate that T-lymphocyte antigen 4 monoclonal antibodies carry a higher risk of gastrointestinal adverse events compared to PD-1/PD-L1 monoclonal antibodies, with occurrences possible during or months after treatment.^[16]^ The median onset time for gastrointestinal adverse events with PD-1/PD-L1 monoclonal antibodies is around 3 months from the start of treatment. Intestinal obstruction, a rare manifestation of immune treatment-related gastrointestinal adverse events, can lead to intestinal perforation and be life-threatening, though reported only in isolated cases. Cho et al^[17]^ reported a case of colonic perforation following nivolumab treatment for esophageal cancer. In the KEYNOTE-177 study, there was a case of intestinal perforation associated with pembrolizumab and chemotherapy combination therapy for colon cancer.^[18]^ Italy documented a case of small intestinal perforation due to pembrolizumab treatment for melanoma metastasis.^[19]^

Currently, there is no clear evidence that the combination of ICIs and anti-angiogenic drugs increases the incidence of adverse reactions. Normann et al^[20]^ identified 1 case of intestinal perforation in their study on the combination of ICIs and bevacizumab, which was attributed to bevacizumab use. However, their study also noted an increasing trend in toxicity with the combination of nivolumab and bevacizumab. Many studies indicate that some serious treatment-related adverse reactions in combined therapy are primarily associated with the addition of anti-angiogenic drugs rather than immune-related adverse reactions caused by ICIs.^[16]^

Based on the findings from the KEYNOTE-826 study,^[21]^ our approach involved administering chemotherapy alongside bevacizumab and pembrolizumab for the treatment of recurrent cervical cancer. Considering the literature and the specific details of this case, where the patient had previously undergone radical radiotherapy and chemotherapy followed by recurrence, the decision was made to combine chemotherapy with anti-angiogenic drugs and ICIs. Unfortunately, after 2 cycles of treatment, the patient experienced intestinal perforation, which aligns more closely with the known risk profile of gastrointestinal perforation associated with bevacizumab. As a result, further treatment was discontinued. The case report we provided also has certain particularity, because this case received radiotherapy, chemotherapy, anti-angiogenic therapy and immunotherapy, and the complications occurred were the result of the combination of all treatments, and did not represent a particular treatment. Therefore, it is of great importance for our clinical work to improve the awareness of the potential risks involved after the combination of treatments.

## 4. Conclusion

The combination of immune checkpoint inhibitors and anti-angiogenic drugs has shown significant survival benefits for patients, but it also presents potential treatment-related toxicities. Further research is necessary to thoroughly compare the efficacy and safety of combined regimens with corresponding monotherapy approaches. Gastrointestinal perforation, though rare, remains a potentially fatal adverse reaction associated with these treatments. In the clinical process of combined medication, more attention should be paid to adverse reactions, early identification of severe adverse reactions, and active management.

## Acknowledgments

The author is particularly grateful to Qiuman Wang for the help in manuscript writing.

## Author contributions

**Conceptualization:** Yuanchun Fan, Jiangjing Zhao, Yawei Fu, Chunyang Wang, Hui Zhang.

**Formal analysis:** Yawei Fu.

**Investigation:** Yuanchun Fan, Jiangjing Zhao.

**Resources:** Yuanchun Fan.

**Software:** Yuanchun Fan.

**Writing – original draft:** Yuanchun Fan, Shihao Liu.

**Writing – review & editing:** Yuanchun Fan, Jiahui Yang, Hui Zhang.

## Supplementary Material


