# Supplementary material for: Intestinal perforation in recurrent cervical cancer following bevacizumab and pembrolizumab therapy: A case report
Source: Medicine (Baltimore). 2025 Apr 11;104(15):e40473. doi: 10.1097/MD.0000000000040473 (PMC11999440; doi:10.1097/MD.0000000000040473)
Supplement: Supplementary file 1 [file medi-104-e40473-s001.docx]

**Consolidated Standards of Reporting Trials**

**Flowchart Diagram**
