# Supplementary material for: Intestinal perforation in recurrent cervical cancer following bevacizumab and pembrolizumab therapy: A case report
Source: Medicine (Baltimore). 2025 Apr 11;104(15):e40473. doi: 10.1097/MD.0000000000040473 (PMC11999440; doi:10.1097/MD.0000000000040473)
Supplement: Supplementary file 2 [file medi-104-e40473-s002.docx]

**SUPPLEMENTAL TABLE 1.** Means (Standard Deviation) of Leg Circumferences (centimeters) at Selected Landmarks across Days for Untaped and Taped Legs in each Time Group.

| **Circumference**  **(cm)** | **Leg** | **Time Group** | **n** | **Day 0** | | **Day 1** | | **Day 2** | | **Day 4** | | **Day 6** | | **Day 8** | |
| --- | --- | --- | --- | --- | --- | --- | --- | --- | --- | --- | --- | --- | --- | --- | --- |
|  |  |  |  | **Mean** | **SD** | **Mean** | **SD** | **Mean** | **SD** | **Mean** | **SD** | **Mean** | **SD** | **Mean** | **SD** |
| 10 cm Above Superior Pole of Patella | Untaped | 6 d or less | 27 | 53.9 | 7.0 | 52.7 | 7.4 | 53.5 | 7.9 | 52.6 | 7.3 | 52.1 | 7.4 | 51.8 | 7.2 |
|  |  | 7 d or more | 25 | 51.8 | 7.5 | 51.0 | 7.0 | 51.9 | 7.4 | 50.8 | 7.6 | 50.1 | 8.7 | 49.4 | 7.9 |
|  |  | Total | 52 | 52.9 | 7.3 | 51.8 | 7.2 | 52.7 | 7.6 | 51.8 | 7.5 | 51.2 | 8.0 | 50.6 | 7.6 |
|  | Taped | 6 d or less | 27 | 54.1 | 7.2 | 53.6 | 7.2 | 53.8 | 7.2 | 52.9 | 7.6 | 52.6 | 7.5 | 52.3 | 7.5 |
|  |  | 7 d or more | 25 | 51.9 | 7.3 | 51.1 | 7.0 | 51.6 | 7.2 | 51.1 | 7.1 | 50.4 | 8.0 | 49.7 | 7.7 |
|  |  | Total | 52 | 53.0 | 7.2 | 52.4 | 7.1 | 52.8 | 7.2 | 52.1 | 7.3 | 51.6 | 7.8 | 51.1 | 7.6 |
| Middle of the Knee Joint | Untaped | 6 d or less | 27 | 45.8 | 6.1 | 46.0 | 6.0 | 46.1 | 5.8 | 45.5 | 5.1 | 45.2 | 4.9 | 45.4 | 5.3 |
|  |  | 7 d or more | 25 | 46.4 | 5.9 | 45.5 | 4.1 | 45.9 | 5.2 | 45.2 | 5.1 | 44.6 | 5.8 | 44.5 | 5.4 |
|  |  | Total | 52 | 46.1 | 5.9 | 45.8 | 5.1 | 46.0 | 5.4 | 45.3 | 5.1 | 44.9 | 5.3 | 45.0 | 5.3 |
|  | Taped | 6 d or less | 27 | 46.3 | 6.0 | 46.4 | 6.3 | 46.4 | 6.2 | 45.7 | 5.6 | 45.6 | 5.3 | 45.6 | 4.9 |
|  |  | 7 d or more | 25 | 45.6 | 4.7 | 45.3 | 4.1 | 45.3 | 4.1 | 44.9 | 4.1 | 44.3 | 4.9 | 43.8 | 4.9 |
|  |  | Total | 52 | 46.0 | 5.4 | 45.9 | 5.3 | 45.8 | 5.3 | 45.3 | 4.9 | 45.0 | 5.1 | 44.8 | 4.9 |
| 3 inches Below Fibular Head | Untaped | 6 d or less | 26 | 41.0 | 5.4 | 41.0 | 5.4 | 40.6 | 5.4 | 40.6 | 5.7 | 40.3 | 5.2 | 40.0 | 5.5 |
|  |  | 7 d or more | 24 | 41.1 | 4.0 | 41.1 | 4.0 | 40.6 | 4.4 | 39.9 | 4.4 | 39.3 | 4.5 | 39.1 | 4.6 |
|  |  | Total | 50 | 41.0 | 4.7 | 41.0 | 4.7 | 40.6 | 4.9 | 40.3 | 5.1 | 39.8 | 4.9 | 39.6 | 5.0 |
|  | Taped | 6 d or less | 26 | 41.2 | 5.1 | 41.2 | 5.1 | 40.6 | 5.0 | 41.0 | 6.0 | 40.0 | 5.5 | 40.1 | 5.1 |
|  |  | 7 d or more | 24 | 40.4 | 3.9 | 40.4 | 3.9 | 40.1 | 4.0 | 39.6 | 4.0 | 39.2 | 4.4 | 38.8 | 4.5 |
|  |  | Total | 50 | 40.8 | 4.6 | 40.8 | 4.6 | 40.3 | 4.5 | 40.3 | 5.1 | 39.6 | 4.9 | 39.5 | 4.8 |
| Figure-8 of Ankle and Foot | Untaped | 6 d or less | 27 | 54.3 | 4.6 | 53.5 | 4.9 | 54.4 | 4.3 | 54.7 | 4.9 | 54.8 | 4.4 | 54.5 | 5.1 |
|  |  | 7 d or more | 25 | 54.8 | 4.2 | 54.6 | 4.6 | 55.5 | 4.8 | 55.3 | 4.4 | 55.1 | 4.5 | 54.6 | 4.9 |
|  |  | Total | 52 | 54.6 | 4.4 | 54.0 | 4.7 | 54.9 | 4.6 | 55.0 | 4.7 | 55.0 | 4.4 | 54.5 | 4.9 |
|  | Taped | 6 d or less | 27 | 54.8 | 4.8 | 54.1 | 4.9 | 54.6 | 4.4 | 55.0 | 5.1 | 54.8 | 4.3 | 54.5 | 4.6 |
|  |  | 7 d or more | 25 | 55.1 | 4.3 | 54.9 | 4.7 | 55.2 | 5.1 | 54.1 | 4.8 | 54.7 | 4.5 | 54.2 | 4.5 |
|  |  | Total | 52 | 54.9 | 4.5 | 54.5 | 4.8 | 54.9 | 4.7 | 54.6 | 4.9 | 54.8 | 4.4 | 54.3 | 4.5 |
| SD = Standard Deviation; cm = Centimeters; d = days  Figure-8 = Measuring tape ran from lateral malleolus to navicular tuberosity, under the plantar aspect of the foot to the tuberosity of the fifth metatarsal to the medial malleolus and posteriorly to the lateral malleolus. | | | | | | | | | | | | | | | |
