# Supplementary material for: Intestinal perforation in recurrent cervical cancer following bevacizumab and pembrolizumab therapy: A case report
Source: Medicine (Baltimore). 2025 Apr 11;104(15):e40473. doi: 10.1097/MD.0000000000040473 (PMC11999440; doi:10.1097/MD.0000000000040473)
Supplement: Supplementary file 3 [file medi-104-e40473-s003.docx]

**SUPPLEMENTAL TABLE 2.** Means (Standard Deviation) of Active and Active-assistive Knee Range of Motion (degrees) across Days for Untaped and Taped Legs in each Time Group.

| **Range of Motion (degrees)** | **Leg** | **Time Group** | **n** | **Day 0** | | **Day 1** | | **Day 2** | | **Day 4** | | **Day 6** | | **Day 8** | |
| --- | --- | --- | --- | --- | --- | --- | --- | --- | --- | --- | --- | --- | --- | --- | --- |
|  |  |  |  | **Mean** | **SD** | **Mean** | **SD** | **Mean** | **SD** | **Mean** | **SD** | **Mean** | **SD** | **Mean** | **SD** |
| Active Knee Flexion | Untaped | 6 d or less | 27 | 69.5 | 13.4 | 75 | 14.2 | 79.6 | 12.8 | 83.3 | 13 | 85.5 | 12.9 | 88.4 | 12.7 |
|  |  | 7 d or more | 25 | 68 | 19.6 | 72.7 | 15.8 | 79.4 | 12.7 | 81.8 | 15 | 85.3 | 12.7 | 89.8 | 12.7 |
|  |  | Total | 52 | 68.8 | 16.5 | 73.9 | 14.9 | 79.5 | 12.6 | 82.6 | 13.9 | 85.4 | 12.6 | 89.1 | 12.6 |
|  | Taped | 6 d or less | 27 | 66.9 | 16.3 | 72.5 | 15.6 | 78.1 | 14.1 | 82.2 | 14.5 | 85.6 | 13.1 | 89.1 | 11.2 |
|  |  | 7 d or more | 25 | 74.9 | 17.8 | 77.7 | 13.9 | 81.8 | 13.5 | 84.2 | 12.1 | 86.6 | 14 | 88.1 | 14 |
|  |  | Total | 52 | 70.8 | 17.3 | 75 | 14.9 | 79.9 | 13.8 | 83.2 | 13.3 | 86.1 | 13.4 | 88.6 | 12.5 |
| Active-assistive  Knee Flexion | Untaped | 6 d or less | 27 | 77.7 | 13.5 | 80.9 | 13.4 | 85.3 | 10.9 | 89 | 12.4 | 90.6 | 11.3 | 93.6 | 12 |
|  |  | 7 d or more | 25 | 75.5 | 18.2 | 81.2 | 14.1 | 86 | 11.4 | 88.4 | 13.4 | 91.9 | 12 | 95.6 | 11.3 |
|  |  | Total | 52 | 76.7 | 15.8 | 81 | 13.6 | 85.7 | 11 | 88.7 | 12.8 | 91.2 | 11.6 | 94.6 | 11.6 |
|  | Taped | 6 d or less | 27 | 74.3 | 14.6 | 81.4 | 13.3 | 85 | 11 | 88.1 | 13.8 | 90.4 | 11.6 | 94.1 | 10.2 |
|  |  | 7 d or more | 25 | 77.6 | 21.9 | 85.9 | 12.6 | 88.6 | 12.1 | 90.7 | 10.7 | 93.2 | 13.4 | 94.9 | 13.2 |
|  |  | Total | 52 | 75.9 | 18.4 | 83.5 | 13 | 86.8 | 11.6 | 89.3 | 12.4 | 91.8 | 12.4 | 94.5 | 11.6 |
| Active Knee Extension | Untaped | 6 d or less | 26 | -5 | 4.3 | -5.8 | 5.4 | -4.2 | 4.2 | -5.1 | 5.7 | -4.6 | 4.7 | -4 | 3.9 |
|  |  | 7 d or more | 24 | -5.9 | 4.4 | -5.4 | 4.4 | -5.3 | 3.6 | -5.3 | 4.2 | -4.6 | 4.6 | -4 | 3.5 |
|  |  | Total | 50 | -5.4 | 4.3 | -5.6 | 4.9 | -4.7 | 3.9 | -5.2 | 4.9 | -4.6 | 4.6 | -4 | 3.7 |
|  | Taped | 6 d or less | 26 | -7.1 | 4.1 | -6.2 | 4.6 | -5.4 | 3.2 | -5.5 | 4.3 | -5.6 | 3.5 | -4.2 | 3.3 |
|  |  | 7 d or more | 24 | -5.7 | 5.3 | -5.4 | 4.6 | -5.2 | 3.9 | -4.4 | 4 | -4.5 | 4.3 | -3.9 | 3.3 |
|  |  | Total | 50 | -6.4 | 4.7 | -5.8 | 4.6 | -5.3 | 3.6 | -5 | 4.2 | -5.1 | 3.9 | -4.1 | 3.3 |
| Active-assistive  Knee Extension | Untaped | 6 d or less | 26 | -3.9 | 4 | -4.2 | 5.1 | -2.8 | 3.4 | -3.5 | 4.7 | -3.3 | 3.9 | -2.4 | 3 |
|  |  | 7 d or more | 25 | -4.5 | 4 | -3.8 | 3.7 | -3.8 | 3.3 | -4 | 3.5 | -3.4 | 3.5 | -2.8 | 2.8 |
|  |  | Total | 51 | -4.2 | 4 | -4 | 4.4 | -3.3 | 3.3 | -3.8 | 4.1 | -3.3 | 3.7 | -2.6 | 2.9 |
|  | Taped | 6 d or less | 26 | -5.3 | 4.2 | -4.5 | 3.9 | -4 | 2.8 | -3.9 | 3.9 | -4 | 3.1 | -2.6 | 2.6 |
|  |  | 7 d or more | 25 | -4.5 | 4.7 | -3.7 | 3.9 | -3.5 | 3.5 | -3.3 | 3.2 | -3.4 | 3.7 | -2.6 | 2.7 |
|  |  | Total | 51 | -4.9 | 4.4 | -4.1 | 3.9 | -3.8 | 3.1 | -3.6 | 3.5 | -3.7 | 3.4 | -2.6 | 2.6 |
| SD = Standard Deviation  d = days | | | | | | | | | | | | | | | |
