# Supplementary material for: Intestinal perforation in recurrent cervical cancer following bevacizumab and pembrolizumab therapy: A case report
Source: Medicine (Baltimore). 2025 Apr 11;104(15):e40473. doi: 10.1097/MD.0000000000040473 (PMC11999440; doi:10.1097/MD.0000000000040473)
Supplement: Supplementary file 4 [file medi-104-e40473-s004.docx]

**SUPPLEMENTAL TABLE 3.** Means (Standard Deviation) of Numerical Pain Ratings (1-10) across Days for Untaped and Taped Legs in each Time Group.

|  | **Leg** | **Time Group** | **n** | **Day 0** | | **Day 1** | | **Day 2** | | **Day 4** | | **Day 6** | | **Day 8** | |
| --- | --- | --- | --- | --- | --- | --- | --- | --- | --- | --- | --- | --- | --- | --- | --- |
|  |  |  |  | **Mean** | **SD** | **Mean** | **SD** | **Mean** | **SD** | **Mean** | **SD** | **Mean** | **SD** | **Mean** | **SD** |
| Numerical Pain Rating | Untaped | 6 d or less | 27 | 5.3 | 2.1 | 5.6 | 2.6 | 5.0 | 2.8 | 5.2 | 2.1 | 4.6 | 2.3 | 4.4 | 2.4 |
|  |  | 7 d or more | 25 | 4.9 | 2.6 | 4.6 | 2.7 | 4.5 | 2.6 | 4.1 | 2.2 | 4.0 | 2.4 | 3.9 | 2.4 |
|  |  | Total | 52 | 5.1 | 2.3 | 5.1 | 2.7 | 4.8 | 2.7 | 4.7 | 2.2 | 4.3 | 2.3 | 4.1 | 2.4 |
|  | Taped | 6 d or less | 27 | 5.3 | 2.1 | 5.4 | 2.5 | 5.0 | 2.7 | 5.2 | 2.3 | 4.5 | 2.3 | 4.4 | 2.4 |
|  |  | 7 d or more | 25 | 4.7 | 2.6 | 4.4 | 2.7 | 4.6 | 2.7 | 3.9 | 2.3 | 4.0 | 2.4 | 3.9 | 2.4 |
|  |  | Total | 52 | 5.0 | 2.3 | 4.9 | 2.6 | 4.8 | 2.7 | 4.6 | 2.3 | 4.3 | 2.3 | 4.1 | 2.4 |
| SD = Standard Deviation; d = days | | | | | | | | | | | | | | | |
